# *Gynura divaricata* in the Modulation of Glucose and Lipid Metabolic Disorders: Research Advances and Translational Challenges

**DOI:** 10.3390/molecules31172993

**Published:** 2026-08-26

**Authors:** Dudong Wei, Long Chen, Fengfeng Xie, Jiahao Lu, Xiuqi Yu, Chennuo Zeng, Zujun Meng, Miao Zhang, Liba Xu, Hua Zhu

**Affiliations:** 1 Guangxi Key Laboratory of Zhuang and Yao Ethnic Medicine, Guangxi University of Chinese Medicine, Nanning 530200, China; wddwdd1223@163.com (D.W.); chenl2015@gxtcmu.edu.cn (L.C.); 15177143553@163.com (F.X.); lujiahao2022@126.com (J.L.); 15078805315@163.com (X.Y.); zengchunnuo@163.com (C.Z.); mzjmzjss@163.com (Z.M.); 2 University Engineering Research Center of Development and Industrialization of Zhuang and Yao Ethnic Medicinal Materials, Guangxi University of Chinese Medicine, Nanning 530200, China; 3 Department of Analytical Chemistry and Food Chemistry, Nutrition and Bromatology Group, University of Vigo, 32004 Ourense, Spain

**Keywords:** *Gynura divaricata*, disorders of glucose and lipid metabolism, insulin resistance, bioactive constituents, mechanisms of action, translational challenges

## Abstract

Glucose and lipid metabolism disorders are closely associated with type 2 diabetes mellitus (T2DM), obesity, hyperlipidemia, and metabolic dysfunction-associated steatotic liver disease (MASLD). *Gynura divaricata* (L.) DC., traditionally consumed as both a food and folk medicine, has attracted increasing attention for its potential regulatory effects on glucose and lipid metabolism. Existing in vitro and in vivo studies suggest that *G. divaricata* extracts and candidate bioactive constituents, including flavonoids, polysaccharides, phenolic acids, and peptides, may improve metabolic phenotypes such as hyperglycemia, dyslipidemia, and insulin resistance. These effects may involve the regulation of intestinal carbohydrate hydrolysis, insulin signaling, gut microbial homeostasis, pancreatic β-cell function, lipid metabolism, oxidative stress, and chronic inflammation. However, human evidence remains limited, and most studies have investigated compound formulations or combined interventions, which are insufficient to establish the clinical efficacy of *G. divaricata* as a standalone preparation. This review summarizes the major bioactive constituents of *G. divaricata*, reviews the current evidence for its glucose-lowering and lipid-regulating effects and related antioxidant and anti-inflammatory mechanisms, and discusses the limitations of existing research. This study aimed to provide a reference for elucidating the mechanisms by which *G. divaricata* regulates glucose and lipid metabolism and for its future clinical translation.

## 1. Introduction

Disruption of glucose and lipid metabolic homeostasis is commonly accompanied by insulin resistance, chronic low-grade inflammation, oxidative stress, adipose tissue dysfunction and hepatic lipid accumulation. These abnormalities contribute substantially to the development and progression of type 2 diabetes mellitus (T2DM), obesity, hyperlipidemia, and metabolic dysfunction-associated steatotic liver disease (MASLD). Insulin resistance impairs glucose utilization in peripheral tissues and promotes hepatic glucose production. Excessive lipid accumulation and associated inflammatory responses disrupt insulin signaling, reinforcing the reciprocal interaction between impaired glucose metabolism and lipid metabolic dysfunction [1,2,3]. As disorders of glucose and lipid metabolism involve multiple interconnected pathological processes, interventions targeting a single pathway may not adequately address the complexity of these metabolic abnormalities. Consequently, increasing attention has been given to plant-derived natural products that contain diverse chemical constituents and may act on multiple metabolic processes [4,5,6].

*Gynura divaricata* (L.) DC. is a perennial herb belonging to the genus *Gynura* in the family Asteraceae. It is mainly distributed in southern and southwestern China, and has a long history of both dietary use and folk medicinal applications. Traditionally, the plant has been consumed fresh or prepared as herbal tea or soup as part of daily dietary practices. In recent years, increasing attention has been paid to its chemical composition and biological activity. Studies have identified a range of constituents, including flavonoids, polysaccharides, phenolic acids, and bioactive peptides, and reported their potential effects on glucose and lipid metabolism, inflammation, and oxidative stress. Owing to its chemical diversity, *G. divaricata* may influence several pathological processes involved in metabolic dysregulation. However, most available studies have focused on individual constituents or isolated mechanisms, and the chemical basis, principal biological processes, and translational challenges associated with its effects on glucose and lipid metabolism disorders have not yet been systematically evaluated [7]. In addition, pyrrolizidine alkaloids (PAs) and their N-oxides (PANOs) were detected in some *G. divaricata* samples [8,9], indicating that both efficacy and safety should be considered in the development of this plant as a functional food ingredient.

This narrative review summarizes and critically evaluates current research on the potential role of *G. divaricata* in the regulation of glucose and lipid metabolism. Relevant literature was retrieved from PubMed, Web of Science, Scopus, and the China National Knowledge Infrastructure (CNKI), and the search was updated through June 2026. Studies specifically investigating *G. divaricata* were prioritized, whereas studies on other *Gynura* species or related natural products were included only for contextual comparisons. This review focuses on the chemical basis, proposed mechanisms, and translational challenges associated with the metabolic effects of *G. divaricata*. Existing evidence was further assessed in relation to the major pathological processes underlying disorders of glucose and lipid metabolism, with the aim of informing future studies on active constituent identification, mechanistic validation, and safety evaluation.

## 2. Chemical Basis Underlying the Potential Effects of *Gynura divaricata* on Glucose and Lipid Metabolism

The major classes of constituents reported to be associated with the regulation of glucose and lipid metabolism in *Gynura divaricata* include flavonoids, polysaccharides, phenolic acids, and peptides. It should be emphasized that the identification of a chemical constituent does not necessarily establish its role as a glucose-lowering or lipid-regulating active component. Current direct evidence is derived primarily from studies using different types of intervention materials, including *G. divaricata* extracts, total flavonoid fractions, polysaccharide fractions, and selected caffeoylquinic acids and oligopeptides, evaluated in vitro or in animal models. At present, other identified constituents should only be regarded as potential contributors to the chemical basis of the observed effects, as direct functional evidence remains limited. The following sections summarize the principal representative constituents within each chemical class and the available evidence linking them to glucose and lipid metabolism.

### 2.1. Flavonoids

Flavonoids are among the most extensively studied classes of constituents in *Gynura divaricata*, with flavonols and their glycosides representing the predominant compounds reported to date. These compounds share a characteristic C_6_–C_3_–C_6_ skeleton and commonly contain multiple phenolic hydroxyl groups, while some occur naturally in glycosylated forms. To date, 15 flavonoid compounds have been isolated and identified from *G. divaricata* [10,11,12] (Table 1). Representative constituents include quercetin, kaempferol, rutin, isoquercitrin, and astragalin. In animal studies, certain flavonoid preparations or flavonoid-enriched fractions have shown glucose-lowering, lipid-regulating, and antioxidant effects. However, the contribution of individual flavonoids to the biological effects of *G. divaricata* remains unclear.

Flavonoid glycosides derived from other plant species, such as cynaroside, have shown metabolic regulatory activity [13] and may provide a useful reference for studies of flavonoids in *Gynura divaricata*. However, such evidence cannot be used to identify these compounds as the principal bioactive constituents responsible for the beneficial effects of *G. divaricata*. Future studies should integrate quantitative compositional analysis, in vivo exposure assessment, and pharmacological evaluation of individual compounds to clarify the relationship between *G. divaricata*-derived flavonoids and improved glucose and lipid metabolism.

The composition and abundance of flavonoids in *G. divaricata* are influenced by geographical origin, harvest time, processing conditions, and extraction methods. Previous studies have indicated that high-altitude cultivation, specific harvesting periods, and appropriate drying procedures may favor flavonoid accumulation [11,14,15,16]. Alcohol-based extraction is commonly used because it can provide a reasonable balance between extraction efficiency and purity [17]. Within a certain temperature range, increasing the extraction temperature has also been associated with higher flavonoid yields and antioxidant activity [18]. However, most existing studies have relied primarily on the total flavonoid content as the main evaluation index and have not adequately addressed the differences in the stability of individual flavonoids during processing and extraction. Therefore, reporting the total flavonoid content alone is insufficient to explain variations in biological activity among different samples. Notably, current evidence only indicates that geographical origin and harvest time may influence the flavonoid composition and content. Nevertheless, direct evidence linking compositional variation among samples from distinct regions to differences in pharmacological effects is still lacking. A more informative quality control strategy should incorporate the measurement of key individual compounds, their processing stability, and chemical markers that are consistently associated with biological effects.

### 2.2. Polysaccharides

*Gynura divaricata* polysaccharides (GDPs) constitute another important class of bioactive constituents that are widely distributed in the roots, stems, and leaves of the plant. Several neutral polysaccharides, including GDPs-1 and GDPs-2, and an acidic polysaccharide designated as GDPs-3, have been isolated from crude *G. divaricata* polysaccharide preparations. Their monosaccharide composition includes glucose, galactose, arabinose, xylose, rhamnose, mannose, glucuronic acid, and galacturonic acid [19,20,21,22] (Table 2). Because extraction, isolation, purification, and nomenclature vary across studies, similarly named fractions, such as GDPs-1, GDPs-2, and GDPs-3, should not be assumed to represent structurally identical polysaccharides.

Structural characterization studies have shown that the repeating unit in the backbone of GDPs-1 is predominantly composed of α-(1→2)-linked galactose residues, whereas that of GDPs-2 mainly contains α-(1→3)-linked mannose residues. Congo red assays further suggested that these polysaccharides may adopt a triple-helical conformation [23]. In another study, a distinct fraction also designated GDPs-1 had a molecular weight of 55.1 kDa and consisted mainly of glucose, galactose, and arabinose, with a backbone formed predominantly by linked glucose and galactose residues [24]. In addition, the acidic polysaccharide GDPs-3 contains uronic acid residues, including glucuronic acid and galacturonic acid, exhibits a chain-like structure and relatively high water solubility, and has been proposed as one of the candidate constituents contributing to the glucose-lowering effects of *Gynura divaricata* [25].

Structural features, such as molecular weight, monosaccharide composition, and glycosidic linkage patterns, may influence the biological activities of polysaccharides. Some studies have reported stronger glucose-lowering activity in polysaccharide fractions with higher glucuronic acid content [21], while lower-molecular-weight fractions have shown greater antioxidant capacity [26]. However, extraction, isolation, nomenclature, and structural characterization methods differ across studies, and the fine structures of GDPs remain incompletely resolved. Future studies should adopt more standardized structural characterization approaches and further investigate the intestinal fermentation products of GDPs and their subsequent effects on the host.

### 2.3. Phenolic Acids

Phenolic acids represent another important class of constituents in *Gynura divaricata* and have been suggested to contribute to the regulation of glucose and lipid metabolism [27]. The phenolic acid profile of *G. divaricata* is dominated by caffeoylquinic acids, particularly chlorogenic acid and dicaffeoylquinic acid isomers 3,4-, 3,5-, and 4,5-dicaffeoylquinic acid. Structurally, these compounds are characterized by a quinic acid core substituted with one or more caffeoyl groups and contain multiple phenolic hydroxyl moieties. In addition, several benzoic acid-type phenolic acids, hydroxycinnamic acid derivatives, and coumarin-related phenolic compounds have been identified [28,29,30,31,32] (Table 3).

Previous studies have suggested that caffeoylquinic acids possess metabolism-related biological activities and may be associated with the regulation of glucose-metabolizing enzymes, pancreatic β-cell function, and oxidative stress [33]. The antioxidant activities reported for benzoic acid and hydroxycinnamic acid derivatives also suggest that these compounds may contribute to the maintenance of metabolic homeostasis [31]. However, little is currently known about the in vivo exposure to phenolic acids derived from *Gynura divaricata* or the relationship between their systemic levels and glucose-lowering outcomes. Much of the available evidence has been obtained using crude extracts. Phenolic acids may act alongside flavonoids and polysaccharides at different stages of metabolic regulation, making it difficult to determine their independent pharmacological contributions. Therefore, direct evidence defining the specific role of phenolic acids in the overall effects of *G. divaricata* on glucose and lipid metabolism remains limited.

### 2.4. Bioactive Peptides

In addition to flavonoids, polysaccharides, phenolic acids, oligopeptides, and other low-molecular-weight peptides derived from *Gynura divaricata* have attracted increasing attention in recent years. One study reported that *G. divaricata*-derived oligopeptides improved glycemic control by suppressing gluconeogenesis and modulating the gut–brain axis [34], whereas several tripeptides exhibited inhibitory activity against dipeptidyl peptidase IV (DPP-IV) [35]. These findings suggest that the peptide constituents may provide additional mechanistic clues regarding the metabolic effects of *G. divaricata*. However, the available evidence is mainly derived from in vitro activity screening and animal experiments. The relationship between peptide structure and the regulation of glucose and lipid metabolism remains poorly defined, and the actual contribution of these peptides to the overall biological effects of *G. divaricata* is yet to be established directly. Before DPP-IV inhibition or gut–brain axis modulation can be considered plausible mechanisms in vivo, several more fundamental questions need to be addressed, including whether these peptides remain intact during gastrointestinal digestion, in what forms they are absorbed, and whether their systemic exposure reaches concentrations comparable to those required for activity in vitro or in animal models.

## 3. Potential Mechanisms Underlying the Effects of *Gynura divaricata* on Disorders of Glucose and Lipid Metabolism

The metabolic effects reported for *Gynura divaricata* are primarily reflected in improvements in glycemic and lipid-related parameters, often accompanied by altered markers of oxidative stress and inflammation. Evidence from enzyme assays, cell models, and animal studies suggests that these beneficial effects may involve several metabolic processes and different classes of constituents, rather than a single compound acting through one specific target. Current evidence indicates that intestinal carbohydrate digestion and absorption, insulin-associated signaling and glucose utilization, gut microbiota and metabolite regulation, and hepatic lipid and cholesterol metabolism are more directly associated with glucose and lipid homeostasis, whereas modulation of oxidative stress and chronic inflammation may play a supportive role. Nevertheless, the causal hierarchy and temporal relationships among these mechanisms remain unclear. Most evidence is still preclinical, and the links between key bioactive constituents, their direct molecular targets, and clinical efficacy have yet to be fully established.

### 3.1. Glucose-Lowering Effects and Potential Mechanisms

**Figure 1:**
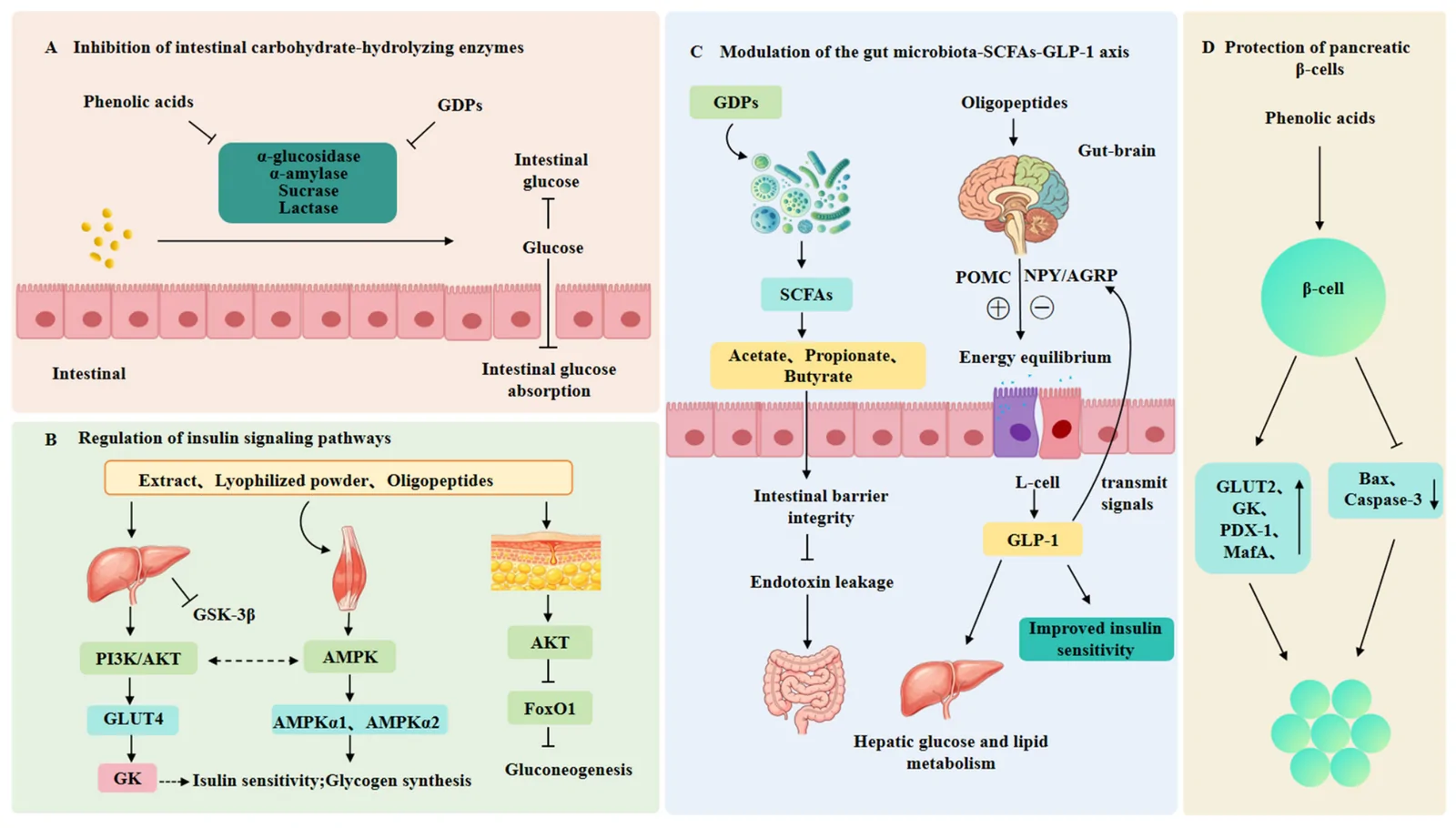
Putative hypoglycemic pathways of *Gynura divaricata* constituents. Current evidence mainly supports effects on intestinal carbohydrate digestion, insulin-related signaling, gut microbiota-derived SCFAs and pancreatic β-cell injury in preclinical models.

Impaired glucose metabolism is a major feature of metabolic disorders involving glucose and lipids. It is associated with several interrelated processes, including intestinal carbohydrate digestion, postprandial glucose absorption, defective insulin signaling, gut microbiota dysbiosis, and impaired pancreatic β-cell function. Glucose-lowering effects of various *Gynura divaricata* extracts, bioactive fractions, and selected constituents have been reported. The proposed mechanisms include delaying intestinal carbohydrate hydrolysis and glucose absorption, improving insulin signaling and glucose utilization, modulating gut microbiota, and preserving pancreatic β-cell function, as summarized in Table 4 and illustrated in Figure 1.

In terms of evidence strength, the most consistent support for the glucose-lowering effects of *Gynura divaricata* currently comes from improvements in the glycemic phenotypes observed in animal models, whereas causal evidence at the level of specific molecular targets remains comparatively limited. In vitro enzyme inhibition studies suggest a potential role in attenuating postprandial increases in blood glucose, while findings from cell-based and animal models support possible improvements in insulin resistance and pancreatic β-cell injury. Clinical evidence remains scarce and is largely derived from studies of compound formulations or combined interventions. Therefore, these findings are insufficient to establish an independent glucose-lowering effect of *G. divaricata* when used as a standalone intervention.

#### 3.1.1. Delayed Intestinal Carbohydrate Hydrolysis and Glucose Absorption

*Gynura divaricata* extracts and certain phenolic acid- and polysaccharide-containing fractions have been reported to inhibit enzymes involved in carbohydrate hydrolysis in vitro and in animal models [28,30,36,37,53]. Among these, *G. divaricata* polysaccharides (GDPs) have been shown not only to directly inhibit α-glucosidase activity but also to modulate the abnormally elevated activities of intestinal disaccharidases, including sucrase, maltase, and lactase, under diabetic conditions. These effects may reduce the conversion of oligosaccharides and disaccharides into absorbable glucose, thereby delaying intestinal glucose absorption [38,39]. Collectively, these findings suggest that *G. divaricata* may attenuate postprandial increases in blood glucose levels by interfering with intestinal carbohydrate digestion. However, the enzyme sources, substrates, and extract compositions varied across studies, and in vitro IC_50_ values cannot be directly extrapolated to glucose-lowering efficacy after oral administration. Moreover, in vitro enzyme inhibition assays do not fully reproduce the complexity of in vivo gastrointestinal digestion and nutrient absorption. Therefore, the current enzyme inhibition data are more appropriately regarded as supportive evidence for a possible mechanism of action rather than as direct evidence of in vivo glucose-lowering efficacy.

#### 3.1.2. Improvement of Insulin Signaling and Glucose Utilization

Cell-based studies have shown that aqueous extracts of *Gynura divaricata* increase glucose consumption and intracellular glycogen content in both HepG2 and insulin-resistant HepG2 cells [40,41]. However, increased glucose consumption and glycogen accumulation in HepG2 cells should only be regarded as preliminary evidence of hepatocyte-like metabolic responses, and cannot substitute for findings obtained from primary hepatocytes or direct assessments of hepatic insulin sensitivity in vivo. Available cellular and animal studies suggest that the glucose-lowering effects of *G. divaricata* extracts and lyophilized preparations of the aerial parts may be associated with the modulation of PI3K/AKT signaling, enhanced GLUT4-mediated glucose uptake, and improved hepatic glucose metabolism. These preparations may promote glycolysis and glycogen synthesis by increasing hepatic glucokinase (GK) expression while suppressing the expression of enzymes involved in gluconeogenesis [41,42,54]. Changes in insulin receptor (InsR)-, AKT-, and glycogen synthase kinase-3β (GSK-3β)-related signaling have also been reported, suggesting a possible involvement in the regulation of glycogen synthesis and insulin sensitivity [55,56,57]. In addition to plant extracts, *G. divaricata*-derived oligopeptides and low-molecular-weight peptides have shown potential glucose-lowering activity. In particular, oligopeptides have been reported to influence FoxO1-mediated transcription of gluconeogenic genes through the AKT/FoxO1 signaling axis [34,35].

In diabetic animal models induced by streptozotocin (STZ), alloxan, or a high-fat/high-sugar diet combined with STZ, *G. divaricata* intervention reduced fasting blood glucose (FBG), glycated hemoglobin (HbA1c), and homeostasis model assessment of insulin resistance (HOMA-IR) while improving glucose tolerance [42,48,49]. STZ- and alloxan-induced models primarily reflect pancreatic β-cell injury, whereas models combining a high-fat/high-sugar diet with STZ reproduce, to some extent, the coexistence of insulin resistance and β-cell dysfunction, which is a hallmark of T2DM. Findings obtained from these different models should therefore not be considered directly equivalent. Lyophilized *G. divaricata* preparations, polysaccharides, total flavonoids, chlorogenic acid, and dicaffeoylquinic acid-related constituents have also been reported to improve glycemic phenotypes in T2DM mouse models [30,38,39,42,43,50]. Several studies have observed changes in proteins associated with AMPK, AKT, and GLUT4 signaling [45,46,47]. At present, however, these alterations are more appropriately regarded as candidate pathways involved in the observed effects than as established causal mechanisms. Most studies on the glucose-lowering effects of *Gynura divaricata* have used extracts or crude fractions, making it difficult to distinguish the contributions of individual bioactive constituents, including polysaccharides, flavonoids, phenolic acids, and peptides. To date, there is insufficient evidence to demonstrate that any specific compound from *G. divaricata* directly activates the PI3K/AKT or AMPK pathway. Whether different bioactive fractions act synergistically, additively, or antagonistically also remains to be experimentally established.

#### 3.1.3. Modulation of the Gut Microbiota

*Gynura divaricata* polysaccharides (GDPs) may act as potential prebiotics and can be utilized by the gut microbiota through fermentation, resulting in the production of short-chain fatty acids (SCFAs), including acetate, propionate, and butyrate. These metabolites may contribute to the regulation of hepatic glucose metabolism and insulin sensitivity [22]. In T2DM models, GDPs have been reported to alter the gut microbial composition, increase SCFA levels, and promote glucagon-like peptide-1 (GLP-1) secretion. These findings suggest that improvements in insulin resistance may involve a regulatory process centered on the polysaccharide–gut microbiota–SCFA–GLP-1 axis [38,44,58].

In addition to polysaccharides, *G. divaricata*-derived oligopeptides have been reported to improve intestinal barrier-related functions, enrich SCFA-producing bacteria, and enhance GLP-1 secretion. These changes may influence the hypothalamic expression of pro-opiomelanocortin (POMC) and neuropeptide Y/agouti-related peptide (NPY/AgRP) through the gut–brain axis, thereby contributing to the regulation of energy homeostasis [34]. However, existing studies have largely focused on associations between gut microbial composition and metabolic phenotypes, with multiple endpoints measured concurrently at the end of the intervention. Causal evidence from approaches such as fecal microbiota transplantation, receptor-specific blockade, or metabolite rescue experiments remains lacking. Direct evidence establishing the causal sequence of polysaccharide exposure to microbial alterations, metabolite production, and subsequent host responses remains limited.

#### 3.1.4. Protection of Pancreatic β-Cells

Available animal studies suggest that certain phenolic constituents of *Gynura divaricata* may exert protective effects against pancreatic β-cell injury. Following intervention with chlorogenic acid- and dicaffeoylquinic acid-related constituents, the expression of genes associated with islet function, including GLUT2, glucokinase (GK), PDX-1, and MafA, increased, whereas the expression of pro-apoptotic proteins such as Bax and caspase-3 decreased. These changes are accompanied by decreased β-cell apoptosis and attenuation of islet structural damage [30]. In addition, aqueous extracts and total flavonoid preparations of *G. divaricata* have been reported to enhance endogenous antioxidant defenses in pancreatic islet cells and alleviate oxidative stress-associated decline in β-cell function [43,51]. However, evidence of the β-cell-protective effects of *G. divaricata* is mainly derived from histopathological observations and protein expression analyses in animal models. Current findings therefore support a potential role in attenuating β-cell injury, but do not establish that *G. divaricata* directly promotes β-cell proliferation or restores β-cell function.

### 3.2. Lipid-Regulating Effects and Potential Mechanisms

**Figure 2:**
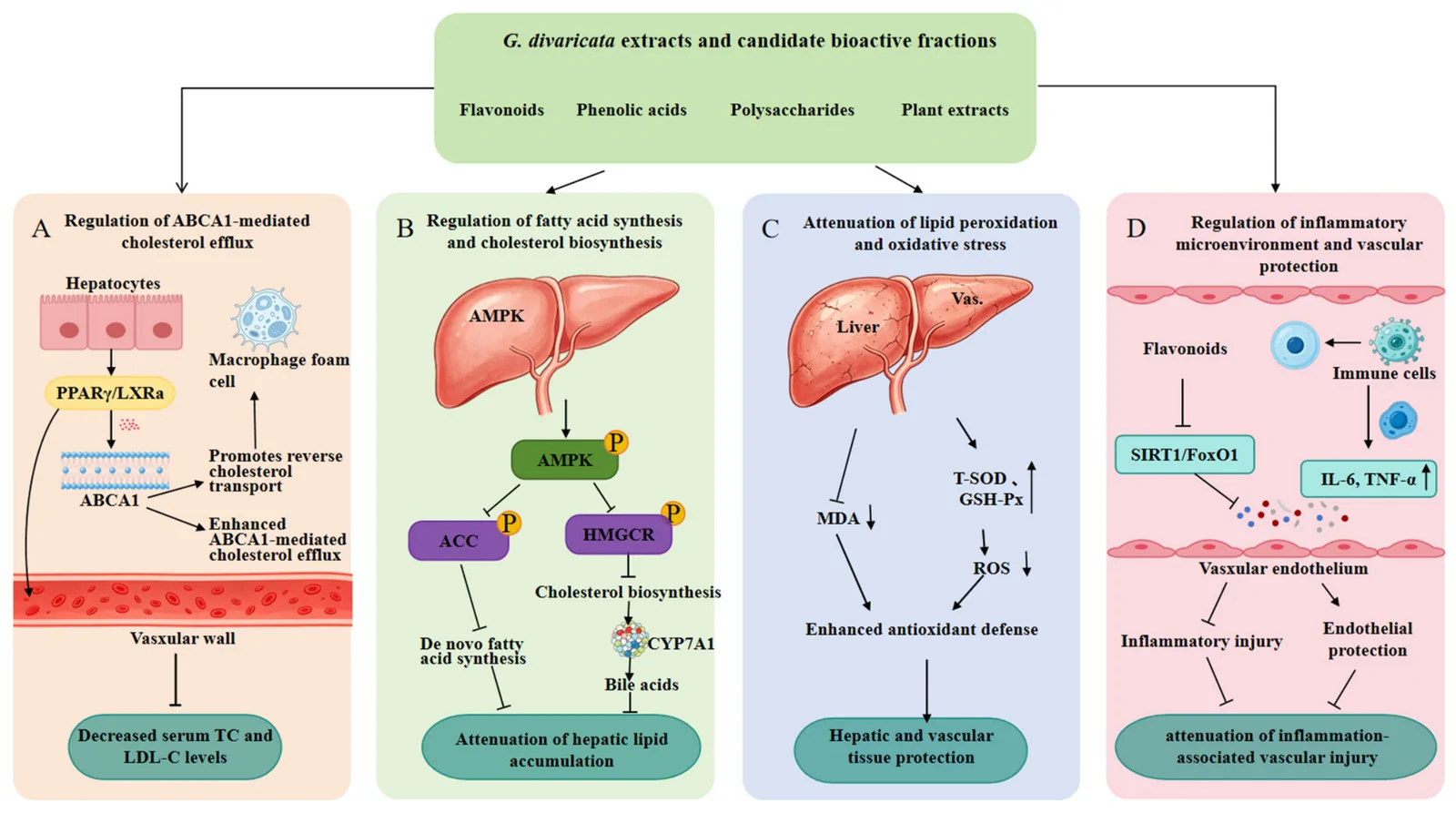
Proposed mechanisms underlying the lipid-regulatory effects of *Gynura divaricata*. Bioactive constituents may promote cholesterol efflux and bile acid synthesis through PPARγ/LXRα/ABCA1 and CYP7A1, inhibit fatty acid and cholesterol biosynthesis through AMPK-mediated regulation of ACC and HMGCR, alleviate lipid peroxidation by strengthening antioxidant defenses, and reduce inflammatory microenvironment-related vascular injury through SIRT1/FoxO1-associated signaling.

Lipid metabolic dysfunction is a major pathological feature of disorders involving glucose and lipid metabolism. Available studies suggest that *Gynura divaricata* has potential lipid-regulating effects in several experimental models of metabolic dysfunction, with most evidence derived from interventions using extracts, total flavonoid preparations, or plant powders. Reported effects include improvements in serum lipid profiles, reduced hepatic lipid accumulation, and attenuation of histopathological tissue damage. These changes may involve the regulation of cholesterol homeostasis, fatty acid synthesis, inflammation, and oxidative stress, as summarized in Table 5 and illustrated in Figure 2. Compared to research on its glucose-lowering effects, evidence concerning the lipid-regulating activity of *G. divaricata* remains relatively limited and is predominantly based on animal studies. Human studies using standardized extracts are lacking, and clear dose–response relationships have yet to be established.

#### 3.2.1. Improvement of Serum Lipid Profiles and Hepatic Lipid Accumulation

In mouse models of obesity-associated T2DM and rat models characterized by insulin resistance and T2DM, *Gynura divaricata* intervention was associated with reduced serum levels of total cholesterol (TC), triglycerides (TG), and low-density lipoprotein cholesterol (LDL-C), together with increased high-density lipoprotein cholesterol (HDL-C) levels. These changes are accompanied by reduced hepatic lipid accumulation and attenuation of hepatocellular steatosis [47,59,60,61,62]. Some studies have also reported less severe aortic lesions in diabetic rats following treatment with aqueous extracts of *G. divaricata* [63]. However, this finding requires confirmation through more direct assessments of vascular function and further mechanistic investigations. It should also be noted that the intervention materials, extraction procedures, administered doses, treatment durations, and animal models differed across studies, limiting the direct comparison of the reported lipid-regulating effects.

#### 3.2.2. Regulation of Cholesterol Homeostasis and Fatty Acid Metabolism

The effects of *Gynura divaricata* on lipid metabolism may involve several processes, including cholesterol transport, bile acid metabolism, fatty acid synthesis and oxidation, and cellular energy sensing. With respect to cholesterol transport, the lipid-regulating effects of *G. divaricata* extracts may be associated with PPARγ/LXRα signaling and ATP-binding cassette transporter A1 (ABCA1)-mediated cholesterol efflux, with possible implications for reverse cholesterol transport. Changes in factors involved in bile acid synthesis, including cholesterol 7α-hydroxylase (CYP7A1), further suggest that *G. divaricata* may influence the conversion of cholesterol into bile acids, thereby contributing to reduced tissue cholesterol accumulation [59].

At the energy sensing level, *G. divaricata* intervention has been associated with alterations in AMPK-related signaling and phosphorylation of acetyl-CoA carboxylase (ACC) and 3-hydroxy-3-methylglutaryl-CoA reductase (HMGCR). These findings suggest that AMPK-related pathways may participate in the regulation of de novo fatty acid synthesis and cholesterol biosynthesis [47]. In addition, total flavonoid preparations from *G. divaricata* have been reported to affect PPAR-related signaling, which may contribute to the regulation of lipid metabolism and inflammatory responses, and may be associated with improvements in lipid metabolic abnormalities and inflammation-related tissue injury [47,59]. However, much evidence supporting these pathways is derived from network pharmacology, molecular docking, and changes in protein expression. These findings are therefore better regarded as mechanistic hypotheses or candidate pathways, rather than definitive evidence that these molecules are key targets mediating the lipid-regulating effects of *G. divaricata*.

### 3.3. Roles of Oxidative Stress and Inflammation in the Metabolic Effects of Gynura divaricata

Oxidative stress and chronic inflammation are closely associated with several pathological processes, including insulin resistance, dysregulated lipid metabolism, and β-cell dysfunction, and may contribute to the development and progression of glucose and lipid metabolic disorders. Accordingly, modulation of oxidative stress and inflammatory responses may represent one of the mechanisms through which *Gynura divaricata* improves glucose and lipid metabolic disturbances. In vitro studies and model organism experiments have shown that *G. divaricata* polysaccharides and their purified fractions possess free radical-scavenging activity. In *Caenorhabditis elegans*, GDPs improve oxidative stress-related phenotypes and enhance tolerance to heat stress [64]. These findings provide preliminary evidence of the antioxidant potential of *G. divaricata*, but should not be directly interpreted as evidence of therapeutic efficacy in mammalian metabolic disease.

Animal studies have reported changes in the expression of Nrf2 and its downstream antioxidant proteins, including HO-1 and NQO-1, following *G. divaricata* treatment. These changes were accompanied by increased activities of antioxidant enzymes, such as superoxide dismutase (SOD) and glutathione peroxidase (GSH-Px), as well as reduced levels of lipid peroxidation products, such as malondialdehyde (MDA) [60,61,63,65]. With respect to inflammation, *G. divaricata* has also been reported to modulate NF-κB pathway activation and reduce the expression of pro-inflammatory mediators, including TNF-α, IL-6, and IL-1β. These molecular changes occur alongside improvements in inflammatory status, insulin resistance, or inflammation-related tissue injury [66]. In addition, total flavonoid preparations from *G. divaricata* may participate in the regulation of oxidative stress, inflammatory responses, and energy metabolism via SIRT1/FoxO1-related signaling [67].

Current evidence on antioxidant and anti-inflammatory effects mainly shows that changes in these parameters occur alongside improvements in metabolic outcomes, but is insufficient to establish the direction of causality. Existing studies lack pathway-specific inhibition and time-course experiments, which hinders efforts to distinguish upstream from downstream regulatory events or to determine the temporal order and necessity of oxidative stress and inflammatory modulation in glucose and lipid metabolism. Therefore, modulation of oxidative stress and inflammation is better regarded as a complementary explanation for the metabolic effects of *Gynura divaricata* rather than an established core mediating mechanism.

## 4. Translational Challenges and Future Perspectives

The major translational barrier facing *Gynura divaricata* is not the lack of positive efficacy findings, but rather the limited comparability of studies using different raw materials, preparations, and experimental models. Moreover, given that the potential risks associated with PAs and PANOs have not yet been adequately quantified, an immediate priority should not be to propose additional candidate mechanisms. Instead, efforts should focus on establishing reproducible and traceable study materials that allow evaluation of efficacy and safety within the same standardized framework.

### 4.1. Evaluation of Current Evidence and Research Limitations

Taken together, current studies on the glucose-lowering and lipid-regulating effects of *Gynura divaricata* suggest that its metabolic activity may involve multiple classes of constituents and several metabolic processes. Research on phenolic acids has focused mainly on the inhibition of intestinal carbohydrate-hydrolyzing enzymes and the protection of pancreatic islet function. The reported effects of polysaccharides have largely been associated with modulation of the gut microbiota, whereas flavonoids may be linked to changes in insulin signaling, energy metabolism, and inflammatory markers. Oligopeptides have shown potential for DPP-IV inhibition and gut–brain axis modulation. However, these findings were obtained using different preparations, experimental models, and study conditions, and various classes of candidate active constituents have generally been evaluated separately. Therefore, their relative contributions to the overall metabolic effects of *G. divaricata* remain unclear, and direct evidence supporting synergistic interactions among these constituents is lacking.

Network pharmacology and molecular docking analyses identified AKT1, AMPK-related signaling, and TNF-α as potential regulatory nodes [68]. These computational findings are useful primarily for generating mechanistic hypotheses and require further validation through functional experiments in cellular and animal models. Therefore, it is more appropriate to conclude that *G. divaricata* contains multiple classes of constituents with the potential to influence glucose and lipid metabolism through several biological processes, rather than to characterize its effects prematurely as an established multi-target synergistic mechanism.

### 4.2. Insufficient Standardization of Raw Materials and Extracts

Considerable heterogeneity exists in the *Gynura divaricata* materials used across the studies. These materials include fresh or dried samples derived from the leaves, aerial parts, or whole plant as well as water extracts, ethanol extracts, total flavonoid fractions, polysaccharide fractions, and freeze-dried powders. Differences in geographical origin, harvest time, post-harvest processing, and extraction conditions may substantially affect the composition and abundance of flavonoids, polysaccharides, and other bioactive constituents [11,14,15,16,17,18]. Consequently, preparations evaluated in different studies may differ markedly in both chemical composition and biological exposure, making direct comparison and independent replication of pharmacological findings difficult.

Therefore, the lack of adequate standardization of *G. divaricata* materials and extracts remains an important limitation of the current evidence base. This problem largely arises from variations in plant sources, the absence of harmonized extraction procedures, and the widespread use of crude extracts in pharmacological studies. Essential information, including botanical authentication, voucher specimen deposition, harvest period, processing conditions, and extraction procedures, is often incompletely reported. Characterization of *G. divaricata* polysaccharides (GDPs) is inconsistent with respect to molecular weight distribution, monosaccharide composition, uronic acid content, and residual protein levels, further limiting comparisons across studies.

Future investigations should, at a minimum, provide traceable information on sample origin, botanical authentication, preparation procedures, and the major chemical characteristics of the tested material. Pharmacological effects and mechanistic findings can be meaningfully compared across studies only when the identity and composition of the investigated samples are sufficiently defined.

### 4.3. The Bioactive Constituents and Key Molecular Targets Remain Unclear

Although a range of flavonoids, polysaccharides, phenolic acids, and peptides have been identified in *Gynura divaricata*, only a limited number of candidate bioactive entities currently meet the basic requirements of a clearly defined source, quantifiable abundance, demonstrable in vivo exposure, and reproducible association with pharmacological effects. Most available studies have used total flavonoids, crude polysaccharides, or complex extracts as test materials. Therefore, the biological effects observed in these studies may reflect the combined actions of multiple constituents and cannot be reliably ascribed to a single compound. Evidence derived from network pharmacology, molecular docking, and changes in pathway-related protein expression is useful for generating mechanistic hypotheses; however, it does not establish a direct or indispensable target relationship.

Systematic studies capable of distinguishing the independent effects and interactions of different bioactive constituents remain limited, and the major active contributors and their key sites of action have yet to be clearly identified. Pharmacokinetic and tissue-exposure data for the bioactive constituents of *Gynura divaricata* remain limited, making it difficult to accurately assess the actual contributions of parent compounds and their metabolites to the overall pharmacological effects. In particular, direct experimental evidence regarding the in vivo absorption, tissue distribution, and target-organ exposure of G. divaricata-derived flavonoids and phenolic acids is currently lacking. Most reported antioxidant, anti-inflammatory, and metabolic regulatory activities have been observed at concentrations used in vitro, and there is no clear evidence that the corresponding parent compounds or their metabolites reach biologically effective concentrations in target tissues in vivo. This limits the direct extrapolation of in vitro findings to mechanisms underlying in vivo efficacy. For polysaccharides and peptides, systematic evidence is also lacking regarding the bioactive forms that arise following gastrointestinal digestion, absorption, and transformation by the gut microbiota; therefore, inferring their in vivo effects solely from in vitro activity measured without prior digestion has inherent limitations.

It also remains uncertain whether the constituents present in the complex extracts act additively, synergistically, or antagonistically, as direct experimental evidence is lacking. Therefore, future mechanistic research should move beyond the continued accumulation of putative pathways and address several more fundamental questions: which constituents are systemically available, which parent compounds or metabolites are consistently associated with efficacy, and which molecular targets are directly involved in the glucose-lowering or lipid-regulating effects of *G. divaricata*.

### 4.4. High-Quality Clinical Evidence Remains Limited

Compared with the relatively extensive in vitro and animal evidence, clinical evidence supporting the glucose-lowering effects of *Gynura divaricata* remains very limited. One small-scale intervention study reported that after 12 weeks of treatment with a compound preparation containing *G. divaricata*, several indicators related to glycemic control improved in patients with type 2 diabetes mellitus (T2DM), accompanied by reductions in some lipid metabolism parameters [52]. Another study found that the combined use of *G. divaricata* extract powder and a quadruple viable bacterial preparation was associated with a decrease in certain inflammation-related markers in patients with T2DM [69].

However, both studies involved either compound formulations or combined interventions, and several methodological limitations remained, including small sample size, insufficient control design, unclear intervention dosage, and inadequate quality control of the tested preparations. These issues not only weaken the reliability of the available clinical evidence but also make comparison across studies and evidence synthesis more difficult. Therefore, the current evidence is insufficient to determine the independent therapeutic effect of *G. divaricata* or to define its effective intake dose, safe intake range, and target population.

In addition, clinical studies have yet to adopt standardized outcome measures, follow-up periods, or frameworks for safety assessment, and adverse events have been reported to a limited extent. These shortcomings further hinder the objective evaluation of the clinical utility of *Gynura divaricata*. Given the current evidence, well-designed randomized controlled trials using standardized preparations are essential to determine the independent effects of *G. divaricata*, establish an appropriate dose range, and adequately assess its safety.

### 4.5. Potentially Harmful Constituents and the Safety of Long-Term Use

The translational development of *Gynura divaricata* should not focus solely on potentially beneficial constituents; potentially harmful compounds must also be evaluated to ensure that evidence of efficacy is not used as a substitute for a systematic safety assessment. PAs and PANOs have been detected in some *G. divaricata* samples, and their levels may vary with geographical origin, plant part, harvest time, and processing method [8,9]. Although these findings do not demonstrate a clinical risk under customary dietary conditions, a history of traditional consumption cannot replace formal exposure assessment or long-term safety studies. Current research on PAs and PANOs has been largely limited to their detection and quantification, and the relationship between different exposure levels and long-term health risks remains poorly defined. This uncertainty represents an important barrier to the standardized development and wider use of *G. divaricata.* In addition to PAs and PANOs, cerebroside-type secondary metabolites have also been isolated from *Gynura divaricata*, some of which exhibit cytotoxic activity in vitro. However, their in vivo effects remain unclear, and in vitro cytotoxicity should not be directly equated with adverse effects in vivo [70,71]. Nevertheless, the potential health risks associated with these compounds warrant attention when G. divaricata is developed for use in functional foods.

Accordingly, the development of standardized *G. divaricata* products should be supported by quality specifications that allow potentially harmful constituents to be measured, raw materials to be traced, and exposure levels to be controlled. For preparations intended for prolonged use, PA and PANO contents should be quantified according to the characteristics of the starting material, and the estimated daily intake should be reported. Liver function, adverse events, and reasons for withdrawal or discontinuation should also be systematically monitored [72,73]. In addition, the effects of different processing, extraction, and purification procedures on PAs/PANOs levels require further investigation. Both bioactive constituents and potentially hazardous compounds should be incorporated into a comprehensive quality-control framework, while appropriate intervention doses and long-term safety also need to be established to support the standardized development of *Gynura divaricata* as a functional food or nutritional adjunct.

## 5. Conclusions

*Gynura divaricata* has a long history of both dietary and traditional medicinal use and has attracted increasing attention for its potential role in the management of glucose and lipid metabolic disorders. Current preclinical evidence suggests that *G. divaricata* and its flavonoids, polysaccharides, caffeoylquinic acids, and peptide constituents may improve metabolic abnormalities, such as hyperglycemia, dyslipidemia, insulin resistance, and fatty liver. These effects appear to involve several interconnected processes, including intestinal carbohydrate hydrolysis, glucose absorption, maintenance of gut microbial homeostasis, insulin sensitivity, pancreatic β-cell function, hepatic lipid metabolism, oxidative stress, and chronic inflammation. However, substantial heterogeneity exists across studies in terms of plant material, formulation, experimental models, and outcome measures. The principal active constituents and their in vivo exposure profiles have not yet been clearly defined, and most proposed mechanisms lack direct causal validation.

Taken together, the available evidence supports *G. divaricata* as a potentially useful adjunctive resource for the management of glucose and lipid metabolism; however, it is not yet sufficient to justify its use as a standalone clinical treatment. Compared with flavonoids, phenolic acids, and peptides, GDPs are supported by more substantial in vitro and in vivo evidence for their regulatory effects on glucose and lipid metabolism and may therefore be regarded as one group of candidate bioactive constituents warranting particular attention. However, in the absence of parallel comparative studies of different fractions, the relative contribution of GDPs to the overall pharmacological effects and the potential synergistic interactions among different constituents remain unclear. Further translation is limited by the lack of standardized preparations, clearly identified bioactive constituents, established effective doses in humans, long-term safety data, and high-quality clinical evidence. Future studies should strengthen the quality control of both raw materials and finished preparations, characterize the in vivo exposure of active constituents and potentially harmful compounds, such as PAs/PANOs, and assess efficacy, safety, and dose–response relationships in rigorously designed human intervention studies. Such work will be essential to provide a more reliable scientific basis for the standardized development of *G. divaricata* as a functional food ingredient or adjunctive nutritional intervention.

## Figures and Tables

**Table 1:** Flavonoids isolated from *Gynura divaricata*.

No.	Compound Name	Flavonoid Skeleton	Glycosylation Pattern	Ref.
1	Quercetin	Quercetin-type flavonol; 3,5,7,3′,4′-pentahydroxyflavone	—	[10]
2	Kaempferol	Kaempferol-type flavonol; 3,5,7,4′-tetrahydroxyflavone	—	[12]
3	Isoquercitrin	Quercetin-type flavonol	O-β-D-glucose at C-3	[10]
4	Astragalin	Kaempferol-type flavonol	O-β-D-glucose at C-3	[11]
5	Rutin	Quercetin-type flavonol	Rha-(1→6)-Glc at C-3	[12]
6	Kaempferol-3-O-galactoside	Kaempferol-type flavonol	O-β-D-galactose at C-3	[12]
7	Kaempferol-3-O-robinobioside	Kaempferol-type flavonol	Rha-Gal at C-3	[12]
8	Quercetin-3-O-galactoside	Quercetin-type flavonol	O-β-D-galactose at C-3	[12]
9	Quercetin-3-O-rhamnoside	Quercetin-type flavonol	O-α-L-rhamnose at C-3	[10]
10	Kaempferol-3-O-rhamnoside	Kaempferol-type flavonol	O-α-L-rhamnose at C-3	[10]
11	Kaempferol-3,7-di-O-glucoside	Kaempferol-type flavonol	O-β-D-glucose at C-3 and C-7	[12]
12	Kaempferol-3-O-rutinoside-7-O-glucoside	Kaempferol-type flavonol	Rutinose at C-3 and glucose at C-7	[12]
13	Kaempferol-3-O-robinobioside-7-O-glucoside	Kaempferol-type flavonol	Robinobiose at C-3 and glucose at C-7	[12]
14	Quercetin-3,7-di-O-glucoside	Quercetin-type flavonol	O-β-D-glucose at C-3 and C-7	[12]
15	Quercetin-3-O-rutinoside-7-O-glucoside	Quercetin-type flavonol	Rutinose at C-3 and glucose at C-7	[12]

Note: “—” indicates the absence of glycosyl substitution. Glycosylation sites and sugar moieties are presented for glycosidic flavonoids.

**Table 2:** Structural characteristics of polysaccharide fractions isolated from *Gynura divaricata*.

No.	Polysaccharide Fraction/Study-Specific Name	Plant Material/Extraction Method	Monosaccharide Composition/Structural Units	Molecular Weight	Structural Features	Ref.
1	GDPs-1 (Liu et al., 2011)	Leaves of *G. divaricata*; water extraction and alcohol precipitation	Not fully specified	1.06 × 10^4^ Da	Neutral polysaccharide with α-glycosidic configuration	[19]
2	GDPs-2 (Liu et al., 2011)	Leaves of *G. divaricata*; water extraction and alcohol precipitation	Not fully specified	4.17 × 10^3^ Da	Neutral polysaccharide with α-glycosidic configuration	[19]
3	GDPs-3 (Liu et al., 2011)	Leaves of *G. divaricata*; water extraction and alcohol precipitation	Not fully specified	3.74 × 10^3^ Da	Acidic polysaccharide with α-glycosidic configuration	[19]
4	GDPs-2 (Wang et al., 2015)	*G. divaricata* polysaccharide fraction	GlcA and Xyl; molar ratio, 1.10:0.63	2.03 × 10^5^ Da	Acidic polysaccharide with α-glycosidic linkages	[21]
5	GDPs-3 (Wang et al., 2015)	*G. divaricata* polysaccharide fraction	Rha, GlcA, Gal, Xyl, and GalA; molar ratio, 0.32:6.00:0.21:1.75:4.30	4.29 × 10^5^ Da	Uronic-acid-rich acidic heteropolysaccharide with α-glycosidic linkages	[21]
6	GDP1 (Shang et al., 2026)	Optimized *G. divaricata* polysaccharide extract	Not fully specified	Not reported	Homogeneous polysaccharide fraction	[20]
7	GDP (Yu et al., 2026)	*G. divaricata* polysaccharide obtained by Vc/H_2_O_2_-assisted extraction	Mainly Gal, Xyl, and Ara, with minor Rha, GalA, GlcA, Glc, Fuc, and Man	15.75 kDa	Low-molecular-weight heteropolysaccharide with favorable viscoelastic properties	[22]
8	GDP1 (Yu et al., 2026)	Purified fraction from Vc/H_2_O_2_-assisted GDP	Mainly Gal, Xyl, and Ara, with minor acidic and neutral monosaccharides	9.83 kDa	Low-molecular-weight purified polysaccharide fraction	[22]
9	GDP3 (Yu et al., 2026)	Purified fraction from Vc/H_2_O_2_-assisted GDP	Mainly Gal, Xyl, and Ara, with minor acidic and neutral monosaccharides	8.04 kDa	Low-molecular-weight purified polysaccharide fraction	[22]

Note: Because similar fraction names have been used in different studies, study-specific names were retained to avoid ambiguity. GlcA, glucuronic acid; GalA, galacturonic acid; Rha, rhamnose; Gal, galactose; Xyl, xylose; Ara, arabinose; Glc, glucose; Man, mannose; Fuc, fucose. “Not reported” indicates that the information was unavailable in the cited study.

**Table 3:** Phenolic acids and related phenolic constituents identified in *Gynura divaricata*.

No.	Compound Name	Structural Class	Structural Feature	Plant Material/Fraction	Identification Status	Ref.
1	Chlorogenic acid	Mono-caffeoylquinic acid	Caffeoyl moiety esterified with quinic acid	70% methanol extract; EtOAc/BuOH fractions	Isolated, identified, and/or quantified	[28,29,31]
2	Neochlorogenic acid	Mono-caffeoylquinic acid isomer	Positional isomer of caffeoylquinic acid	Aerial parts/phenolic fractions	Tentatively identified	[32]
3	Cryptochlorogenic acid	Mono-caffeoylquinic acid isomer	Positional isomer of caffeoylquinic acid	Aerial parts/phenolic fractions	Tentatively identified	[32]
4	3,4-Dicaffeoylquinic acid	Dicaffeoylquinic acid	Two caffeoyl moieties esterified at C-3 and C-4 of quinic acid	EtOAc fraction; methanol extract; n-BuOH fraction	Isolated, identified, and/or quantified	[29,31]
5	3,5-Dicaffeoylquinic acid	Dicaffeoylquinic acid	Two caffeoyl moieties esterified at C-3 and C-5 of quinic acid	Leaves; EtOAc fraction; methanol extract	Isolated, identified, and/or quantified; reported as a major compound	[29,31]
6	4,5-Dicaffeoylquinic acid	Dicaffeoylquinic acid	Two caffeoyl moieties esterified at C-4 and C-5 of quinic acid	EtOAc fraction; methanol extract; n-BuOH fraction	Isolated, identified, and/or quantified	[29,31]
7	Salicylic acid	Hydroxybenzoic acid derivative	ortho-Hydroxybenzoic acid scaffold	Aerial parts/phenolic fractions	Reported or tentatively identified	[32]
8	Isovanillic acid	Methoxy-hydroxybenzoic acid derivative	Methoxylated hydroxybenzoic acid scaffold	Ethyl acetate fraction of aerial parts	Isolated and identified	[29,30]
9	p-Coumaric acid	Hydroxycinnamic acid derivative	C6-C3 phenylpropanoid scaffold	Ethyl acetate fraction of aerial parts	Isolated and identified	[29,30]
10	Esculetin	Coumarin-type phenolic compound	6,7-Dihydroxycoumarin scaffold	Ethyl acetate fraction of aerial parts	Isolated and identified	[29,30]

Note: EtOAc, ethyl acetate; BuOH, n-butanol. As the nomenclature of mono-caffeoylquinic acid isomers may vary according to the quinic acid numbering system, the names used in the original references should be retained when discussing individual studies.

**Table 4:** Hypoglycemic effects and putative mechanisms of *Gynura divaricata*.

No.	Model	Active Component/Preparation	Main Findings	Mechanistic Relevance and Evidence Gap	Ref.
1	Cell-free digestive enzyme inhibition assays	Water extract and polarity fractions of *G. divaricata*	Inhibited α-amylase, α-glucosidase, and angiotensin-converting enzyme activities	Provides biochemical support for delayed carbohydrate digestion. The metabolic significance of ACE inhibition remains peripheral to the antidiabetic evidence and requires independent validation.	[35]
2	In vitro α-glucosidase inhibition assay	*G. divaricata* extract	Inhibited α-glucosidase in a concentration-dependent manner, with an IC_50_ of 64.49 μg/mL, close to acarbose IC_50_ = 52.55 μg/mL	Supports a potential postprandial glucose-lowering effect, but intestinal exposure, bioavailability, and in vivo efficacy are not established by this assay alone.	[36,37]
3	In vitro enzyme inhibition and binding-related studies	Caffeoylquinic acid derivatives	Showed α-glucosidase inhibitory activity	Structure-activity observations implicate caffeoyl substitution pattern, caffeoyl group number, and methylation status. Binding-site conclusions remain mainly supportive rather than causal.	[28,33]
4	STZ-induced diabetic rats and intestinal disaccharidase activity assays	*G. divaricata* polysaccharides, GDPs	Suppressed α-glucosidase activity and corrected abnormal sucrase, maltase, and lactase activities under diabetic conditions	Links GDPs to reduced intestinal conversion of oligo- and disaccharides into absorbable glucose. Direct evidence for altered intestinal glucose absorption remains limited.	[38,39]
5	HepG2 cells and insulin-resistant HepG2 cells	Water extract or polysaccharide fraction of *G. divaricata*	Increased glucose consumption and intracellular glycogen content	Cellular data are consistent with improved hepatocyte-like glucose utilization and glycogen storage. Confirmation in primary hepatocytes and in vivo liver tissue would strengthen this interpretation.	[40,41]
6	High-fat diet/STZ-induced T2DM mice; dietary intervention for 4 weeks	Lyophilized powder of *G. divaricata* leaves and stems	Reduced fasting blood glucose and insulin resistance-related indices; increased hepatic glycogen synthesis and antioxidant enzyme activities	Metabolic recovery was accompanied by changes in PI3K/AKT-related and antioxidant markers, supporting, but not proving, improved insulin signaling and oxidative stress control.	[42,43]
7	STZ-induced T2DM mice and insulin-resistant HepG2 cells	GDPs or optimized GDP fraction	Improved fasting blood glucose, oral glucose tolerance, serum insulin, hepatic glycogen, and glucose and lipid metabolism-related indices	Coordinated changes in PI3K/Akt, AMPK, GS/GSK-3β, and GLUT4-related markers place GDPs within insulin signaling and energy metabolism networks. Pathway dependence still requires direct intervention studies.	[20,38,44]
8	High-fat/high-sugar diet plus STZ-induced T2DM rats; extract intervention at 0.5–2.0 g/kg for 4 weeks	Aqueous extract of *G. divaricata*	Reduced fasting plasma glucose and improved diabetes-related biochemical abnormalities	Upregulation of PI3K p85, p-AKT, GLUT4, AMPK/p-AMPK, PPARα, and CPT1α connects the extract with hepatic insulin signaling and fatty acid metabolism. Causal pathway blockade has not yet been shown.	[45,46,47]
9	High-fat diet/STZ-induced T2DM mice; dietary intervention for 4 weeks	*G. divaricata* powder rich in chlorogenic acid and dicaffeoylquinic acids	Reduced FBG, fasting serum insulin, and glycosylated serum protein; improved pancreatic islet morphology	The β-cell phenotype is supported by higher GLUT2, GK, PDX-1, MafA, and Bcl-2 expression and lower Bax and caspase-3 expression, consistent with preserved β-cell function and reduced apoptosis.	[30]
10	STZ/HFD-induced diabetic mice	*G. divaricata*-derived oligopeptides	Improved hyperglycemia, dyslipidemia, insulin resistance, hepatic glycogen synthesis, and pancreatic apoptosis	AKT/FoxO1-related changes support reduced gluconeogenic signaling. Antibiotic-depletion experiments strengthen the microbiota-related interpretation, although clinical translation remains untested.	[34]
11	In vitro DPP-IV inhibition assay	*G. divaricata*-derived tripeptides	Exhibited DPP-IV inhibitory activity	Provides a candidate incretin-related mechanism. In vivo DPP-IV inhibition, GLP-1 response, and glucose-lowering efficacy remain to be demonstrated.	[35]
12	T2DM mice and related diabetic animal models	Fresh material, dried material, lyophilized powder, polysaccharides, total flavonoids, chlorogenic acid, and dicaffeoylquinic acid derivatives	Reduced FBG, HbA1c, HOMA-IR, and glucose intolerance; alleviated pancreatic islet injury; fresh material showed stronger activity than dried material	The preparation-level pattern supports hypoglycemic potential across multiple intervention forms. However, formulation heterogeneity limits attribution to specific constituents or shared mechanisms.	[30,38,39,42,43,48,49,50]
13	Network pharmacology combined with limited experimental validation	Multiple components of *G. divaricata*	PI3K, AKT, AMPK, and GLUT4 were identified as candidate regulatory nodes	Useful for target prioritization, but network-based predictions should be treated as hypothesis-generating unless supported by direct experimental validation.	[46,47]
14	In vitro fermentation and T2DM mouse models with gut microbiota analysis	GDPs	Were utilized by gut microbiota; increased SCFAs, including acetate, propionate, and butyrate; modulated gut microbiota composition and promoted GLP-1 secretion	The parallel changes in microbiota, SCFAs, and GLP-1 support a microbiota-associated metabolic mechanism. Causality would be stronger with depletion, fecal transfer, receptor blockade, or metabolite rescue experiments.	[22,38,44]
15	STZ/HFD-induced diabetic mice with antibiotic-depletion validation	*G. divaricata*-derived oligopeptides	Repaired intestinal barrier, enriched SCFA-producing bacteria, promoted GPR43-dependent GLP-1 secretion, and modulated hypothalamic POMC and NPY/AgRP expression	Provides comparatively stronger support for a gut microbiota-GLP-1-brain axis than association-only studies. Human validation is still lacking.	[34]
16	Diabetic animal models and islet oxidative injury-related models	Water extract and total flavonoids of *G. divaricata*	Enhanced endogenous antioxidant defense in pancreatic islet cells and attenuated β-cell functional decline	β-cell protection is consistent with antioxidant activity, but dependence on oxidative-stress pathways has not been directly established.	[43,51]
17	Small-sample clinical intervention in T2DM patients	Compound preparation containing *G. divaricata*	A 12-week intervention improved glycemic control-related indices and decreased selected lipid metabolism-related parameters	Clinical relevance is suggested, but the combined-intervention design prevents attribution of efficacy or mechanism to *G. divaricata* alone.	[52]

Abbreviations: ACE, angiotensin-converting enzyme; AKT/PKB, protein kinase B; AMPK, AMP-activated protein kinase; DPP-IV, dipeptidyl peptidase-IV; FBG, fasting blood glucose; GDPs, *Gynura divaricata* polysaccharides; GK, glucokinase; GLP-1, glucagon-like peptide-1; GLUT2/4, glucose transporter 2/4; GSK-3β, glycogen synthase kinase-3β; HbA1c, glycated hemoglobin; HFD, high-fat diet; HOMA-IR, homeostasis model assessment of insulin resistance; PDX-1, pancreatic and duodenal homeobox 1; PI3K, phosphoinositide 3-kinase; POMC, pro-opiomelanocortin; SCFAs, short-chain fatty acids; STZ, streptozotocin; T2DM, type 2 diabetes mellitus. Note: Evidence was appraised based on the study type, model relevance, preparation standardization, and depth of mechanistic validation. Pathway-marker changes were considered supportive but insufficient to establish causal dependence.

**Table 5:** Lipid-lowering effects and putative mechanisms of *Gynura divaricata*.

No.	Model	Active Component/Preparation	Main Findings	Mechanistic Relevance and Evidence Gap	Ref.
1	T2DM mice, T2DM rats, and hyperlipidemia-related animal models	*G. divaricata* extract	Decreased serum TC, TG, and LDL-C levels and increased HDL-C levels	Demonstrates improvement of systemic lipid profiles across diabetic or hyperlipidemic models. Differences in model type and extract composition limit cross-study comparability.	[47,59]
2	Obesity-related T2DM mice and insulin resistance combined with T2DM rats	*G. divaricata* intervention	Reduced hepatic lipid deposition and alleviated liver histopathological injury	Supports attenuation of hepatic steatosis and reduced hepatic lipid accumulation. Direct lipid-flux evidence is still limited.	[59,60,61,62]
3	Diabetes-associated atherosclerotic vascular injury animal model	Water extract of *G. divaricata*	Alleviated diabetes-associated aortic atherosclerotic lesions and related metabolic abnormalities	Extends the metabolic phenotype to vascular protection. Evidence for direct effects on endothelial function, macrophage foam-cell formation, or plaque stability remains insufficient.	[63]
4	High-fat diet-induced dyslipidemic animal model	Total flavonoids of *G. divaricata*	Decreased TC and TG levels and alleviated lipid accumulation-related liver injury	Indicates lipid-lowering and hepatoprotective activity. The contribution of individual flavonoids has not been clearly separated.	[62]
5	Lipid metabolism disorder-related animal model	*G. divaricata* extract	Promoted cholesterol efflux and reduced abnormal cholesterol accumulation in the liver and vascular wall	Changes in PPARγ/LXRα/ABCA1 and CYP7A1-related markers are consistent with enhanced reverse cholesterol transport and bile acid conversion. Functional flux assays are needed to confirm this mechanism.	[59]
6	Network pharmacology combined with lipid metabolism-related experimental models	Total flavonoids or multiple components of *G. divaricata*	Improved lipid metabolism and organ injury-related phenotypes	PPAR family-related signaling was identified as a candidate regulatory module. Without direct target validation, this remains mainly hypothesis-generating.	[47,59]
7	High-fat/high-sugar diet plus STZ-induced T2DM rats and hepatic lipid accumulation-related models	*G. divaricata* extract or active fractions	Reduced lipid accumulation and improved lipid metabolic abnormalities	Altered phosphorylation of AMPK, ACC, and HMGCR is compatible with reduced fatty acid and cholesterol biosynthesis. The absence of lipid synthesis or oxidation flux measurements limits causal interpretation.	[47]

Abbreviations: ABCA1, ATP-binding cassette transporter A1; ACC, acetyl-CoA carboxylase; AMPK, AMP-activated protein kinase; CYP7A1, cholesterol 7α-hydroxylase; HDL-C, high-density lipoprotein cholesterol; HMGCR, HMG-CoA reductase; LDL-C, low-density lipoprotein cholesterol; LXRα, liver X receptor α; PPAR, peroxisome proliferator-activated receptor; STZ, streptozotocin; T2DM, type 2 diabetes mellitus; TC, total cholesterol; TG, triglyceride. Note: Evidence was narratively appraised based on study type, model relevance, preparation standardization, and depth of mechanistic validation. Pathway-marker changes were considered supportive but insufficient to establish causal dependence.

## Data Availability

No new data were created or analyzed in this study. Data sharing is not applicable to this article.
